# A systematic review and quality assessment of economic evaluations of kidney replacement therapies in end-stage kidney disease

**DOI:** 10.1038/s41598-024-73735-8

**Published:** 2024-10-03

**Authors:** Patricia Nyokabi, Sitaporn Youngkong, Bhavani Shankara Bagepally, Tabitha Okech, Usa Chaikledkaew, Gareth J McKay, John Attia, Ammarin Thakkinstian

**Affiliations:** 1 Mahidol University Health Technology Assessment Graduate Program, Bangkok, Thailand; 2 Ministry of Health, Nairobi, Kenya; 3 Social and Administrative Pharmacy Division, Department of Pharmacy, Faculty of Pharmacy, Mahidol University, Bangkok, Thailand; 4 Indian Council of Medical Research (ICMR), National Institute of Epidemiology, Chennai, India; 5 Centre for Public Health, Queen’s University Belfast, Belfast, United Kingdom; 6 Centre for Clinical Epidemiology and Biostatistics, School of Medicine and Public Health, University of Newcastle, Newcastle, NSW Australia; 7 Department of Clinical Epidemiology and Biostatistics, Faculty of Medicine, Ramathibodi Hospital, Mahidol University, Bangkok, Thailand

**Keywords:** End-stage kidney disease, Kidney replacement therapies, Economic evaluation, Systematic review, Health care economics, Health services, Health care

## Abstract

End-stage kidney disease (ESKD) is fatal without treatment by kidney replacement therapies (KRTs). However, access to these treatment modalities can be problematic given the high costs. This systematic review (SR) aims to provide an updated economic evaluation of pairwise comparisons of KRTs and the implications for the proportion of patients with access to the KRT modalities, i.e., kidney transplantation (KT), hemodialysis (HD), and peritoneal dialysis (PD). This SR was conducted in accordance with the Preferred Reporting Items for Systematic Reviews and Meta-Analysis (PRISMA) 2020. We searched studies in PubMed, Embase, Scopus, and Cost Effectiveness Analysis (CEA) registry, from inception to March 2023. Thirteen studies were included with pairwise comparisons among three KRTs, with varying proportions of patients for each modality. Seven studies were from high-income countries, including five from Europe. Summary findings are presented on a cost-effectiveness plane and incremental net benefit (INB). KT was the most cost-effective intervention across the pairwise comparisons. KT and PD were both more cost-effective alternatives to HD. HD was more costly and less effective than PD in all studies except one. Concurrent efforts to increase both KT and PD represented the best scenario to improve treatment options for ESKD patients.

**Supplementary Information:**

The online version contains supplementary material available at 10.1038/s41598-024-73735-8.

## Introduction

End-stage kidney disease (ESKD) is characterized by a progressive irreversible decline in kidney function that requires dialysis or transplantation and it is associated with premature mortality^1,2^. The burden of ESKD continues to increase, affecting approximately 10 million people annually^3^, with a reported global incidence of 144 individuals per million population^4^. The ESKD prevalence is higher in high- and upper-middle-income countries (HIC, upper-MICs), at 0.2% and 0.1%, in contrast to lower-middle and low-income countries (lower-MICs, LICs) at 0.07% and 0.05% respectively^5^.

ESKD prevalence is expected to increase further given the increase in non-communicable diseases (NCDs), e.g., hypertension, diabetes mellitus, cardiovascular diseases, and obesity, all of which are considered risk factors for ESKD, in addition to the increasing age of global populations^3,6–8^. An estimated 14.5 million people are predicted to succumb to ESKD globally by 2030, but only 37% (ca. 5.4 million) are likely to have accessibility to appropriate treatments^9^.

ESKD is managed through the provision of kidney replacement therapies (KRT) including kidney transplantation (KT), peritoneal dialysis (PD) and hemodialysis (HD)^10,11^. There is significant geographical and income-related variation in the provision of KRT, with 96% of ESKD patients from LICs unable to access KRT, compared to 40% of patients in HICs^9^. Although > 80% of dialysis patients in many countries receive HD, PD is still the principal KRT in some countries, (e.g., Hong Kong and Mexico), accounting for about 71% and 61% of dialysis patients, respectively.

KT remains the optimum treatment option for all ages regardless of the underlying cause of ESKD^12,13^, providing improved quality of life and survival length^14^. In the US, adjusted mortality rate was lowest for KT, followed by PD and HD with rates of 29, 154 and 166 per 1000 patient-years, respectively^15^. However, limited access to donor organs, high costs, lack of infrastructure and a shortage of skilled healthcare resources have restricted the use of KT as a therapeutic option; therefore, dialysis is more commonly prescribed^6,16^.

Despite the proven efficacy of all KRTs, patient accessibility across many countries remains problematic^9^. These barriers to access are mostly attributable to costs^17^ with significant implications for health coverage schemes^3^. Evidence from a systematic review to assess the cost-effectiveness of KRTs, particularly in resource limited settings, is required to provide support for health policy decisions. Previous reports were varied in terms of the cost-effectiveness of KRTs across regions and national income levels; for instance, a ‘PD-First’ policy was suggested to be most cost-effective in some studies^18,19^, in contrast to KT in others^10,20^. Most previous SRs focused on economic considerations related to the cost of illness or quality of life^21–23^. Other economic evaluations only focused on one or two KRT modalities^6,24–26^, with only a single study addressing the cost-effectiveness of all three KRTs^27^, albeit with limited ability to combine data.

Therefore, this study was conducted to provide an updated review of the cost-effectiveness of KRT modalities to determine the most cost-effective treatment using incremental net benefits (INB) and incremental cost-effectiveness ratios (ICER)^28^. Negative ICER may imply higher cost and lower intervention effectiveness or lower cost and higher intervention effectiveness leading to some ambiguity in interpretation, in contrast to the INB, which is more informative for the cost-effectiveness of treatment options^29^. In addition, ICER requires a specific threshold for interpretation, unlike the INB which incorporates a threshold within its estimation, providing more robust cost-effectiveness evaluations across studies^30,31^.

## Methods

This study was conducted in accordance with the Preferred Reporting Items for Systematic Reviews and Meta-Analysis (PRISMA) 2020 statement^32^, and the review protocol was registered at PROSPERO (ID CRD42022358152).

### Data sources and search strategy

The following online databases were systematically searched: Medline through PubMed, Embase, Scopus, Cost Effectiveness Analysis (CEA) registry by Tufts Medical Centre, National Health Service-Economic Evaluation Database (NHS-EED), Database of Abstracts of Reviews of Effect (DARE) and International Health Technologies Assessment (HTA) Database from inception to March 2023. The search terms are provided in Supplementary Table S1 and there were no restrictions on countries, regions or language.

Studies were selected if they met the following criteria: included an empirical cost-effectiveness and/or cost-utility analysis (CEA, CUA) in adult ESKD patients, considered any comparison of three KRTs (i.e., KT, HD and PD), and reported any of the following outcomes: ICER, INB, cost per quality-adjusted life year (QALY), cost per life year (LY) gained, incremental cost or incremental effectiveness. Studies were ineligible if they were partial economic evaluation studies, conference abstracts, commentaries, or review studies.

### Selection of studies and data extraction

Two authors (PN and TO) independently selected studies by initially screening titles and abstracts. Full papers were then analyzed if a decision could not be made based on the abstract alone. Extracted data included study characteristics (i.e., study setting, economic evaluation type and design, willingness-to-pay (WTP) threshold, perspective, currency, discount rate, and time horizon), interventions and comparators, the study findings, including costs and utilities of interventions versus comparators, ICER, sensitivity analysis and study conclusions.

Any missing relevant data was sought on at least two occasions from the study’s corresponding authors. If the author did not respond, the article was excluded from the review. As a result, one article was disregarded. Any disagreements were resolved through discussion and consensus with the other authors (BSB, SY, UC, AT).

### Quality assessment

A risk of bias assessment for all included studies was evaluated using the economic evaluation bias (ECOBIAS) checklist^33^ which has two parts: a general bias consisting of eleven items, and a model specification bias which included model structure, data, and consistency. Each item was graded as yes (high risk of bias), no (low risk of bias), partly, unclear or not applicable. Additionally, the completeness of reporting was assessed using the Consolidated Health Economic Evaluation Reporting Standards (CHEERS) checklist^34^ with 28 reference points. PN and TO carried out the quality assessment.

### Data analysis

Cost-effectiveness analysis of KRT were described based on the study ICERs relative to the corresponding WTP threshold for that country. These results were collated as INB, an increment in the number of effectiveness units multiplied by the corresponding country threshold providing a beneficial increase in effectiveness expressed in monetary terms minus an incremental cost^35,36^. An intervention is considered cost-effective when the INB value is positive, i.e., greater than zero^37^. The WTP threshold was set according to the original included study; where not provided, it was calculated as three times the gross domestic product (GDP) per capita for each country, as reported by the World Health Organization (WHO)^38^, for the year in which the study was undertaken, with subsequent adjustment for consumer price index (CPI) and purchasing power parity (PPP)^39^. Studies that only provided standard country-specific thresholds were adjusted for PPP. Additionally, all currency data was harmonized to US$ 2022 using both CPI and PPP^40^; the currency conversion factors are provided in Supplementary Table S4.

Cost-effectiveness findings were summarized in a cost-effectiveness plane represented by incremental QALY on the x-axis and incremental cost on the y-axis. It has four quadrants; points that fell within the upper quadrants were considered more costly, while those in the right quadrants were considered more effective^41^.

Studies considered various KRT scenarios. Therefore, re-classification was undertaken to provide uniformity of KRT modalities to enable comparisons across studies. As a result, 5 scenarios were constructed as follows: Scenario 1 was classified to represent the current KRT status in each country and was used as the comparator. Scenario 2 included an increase in PD coverage with a corresponding decrease in HD, while KT was held constant. Scenario 3 included an increase in KT coverage with a corresponding decrease in HD, while PD was held constant. Scenario 4 included an increase in both PD and KT, with a decrease in HD, and Scenario 5 was any other approach reported and not captured under any of the aforementioned scenarios.

## Results

### Study selection and characteristics

**Fig. 1:**
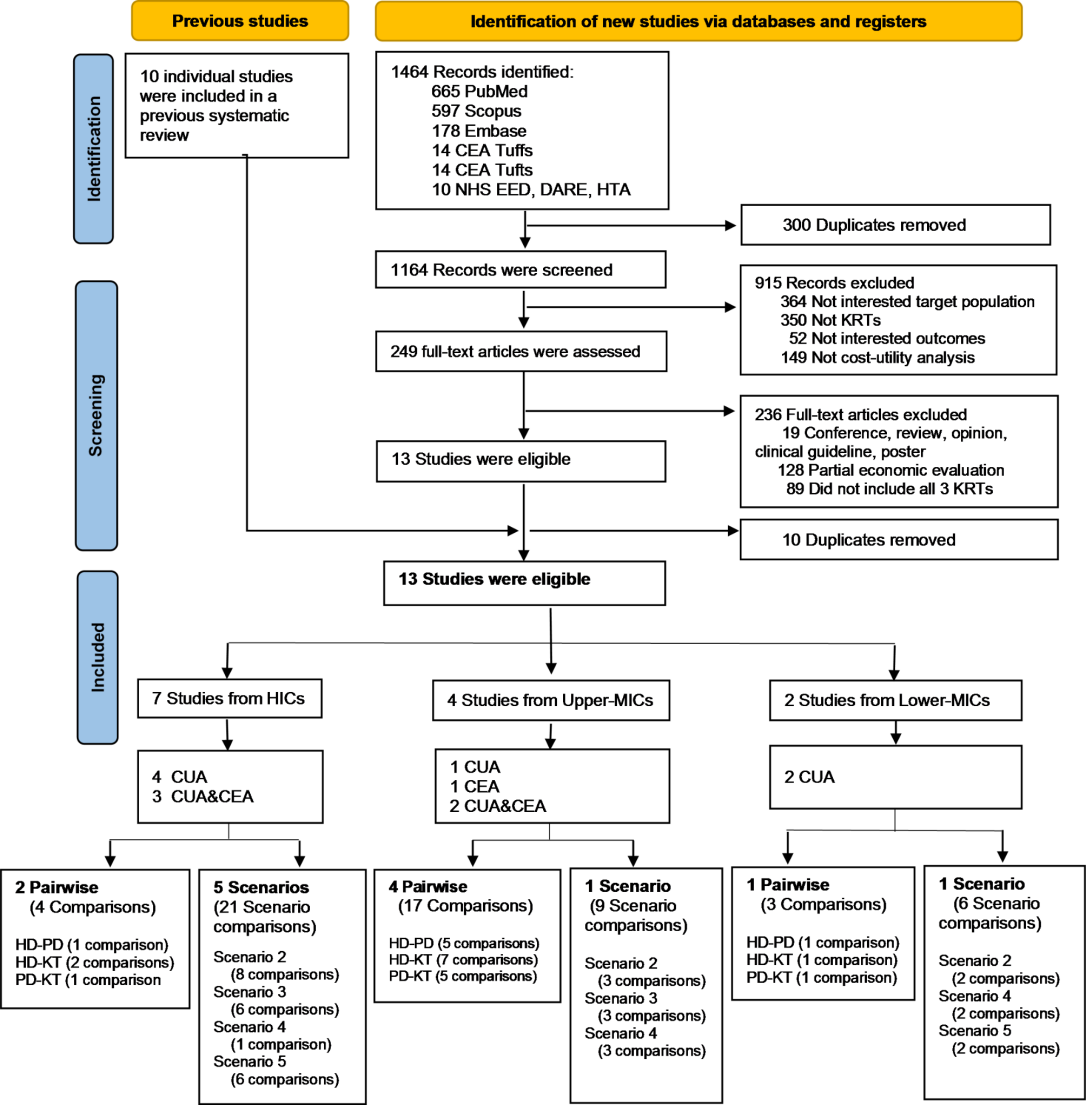
PRISMA flow diagram. CEA, Cost-Effectiveness Analysis; CUA, Cost-Utility Analysis; DARE & HTA, Database of Abstracts of Reviews of Effects and Health Technology Assessment Database; EE, Economic Evaluation, HD, Haemodialysis; HIC, High Income Country; KRT, Kidney Replacement Therapy; KT, Kidney Transplantation; MIC, Middle Income Country; NHS-EED, National Institute of Health Research-Economic Evaluation Database; PD, Peritoneal Dialysis.

There were 1464 articles identified from the systematic search of online databases. Of these, 13 met the inclusion criteria after duplicates and ineligible studies were removed as shown in the PRISMA flow diagram in Fig. 1.

**Fig. 2:**
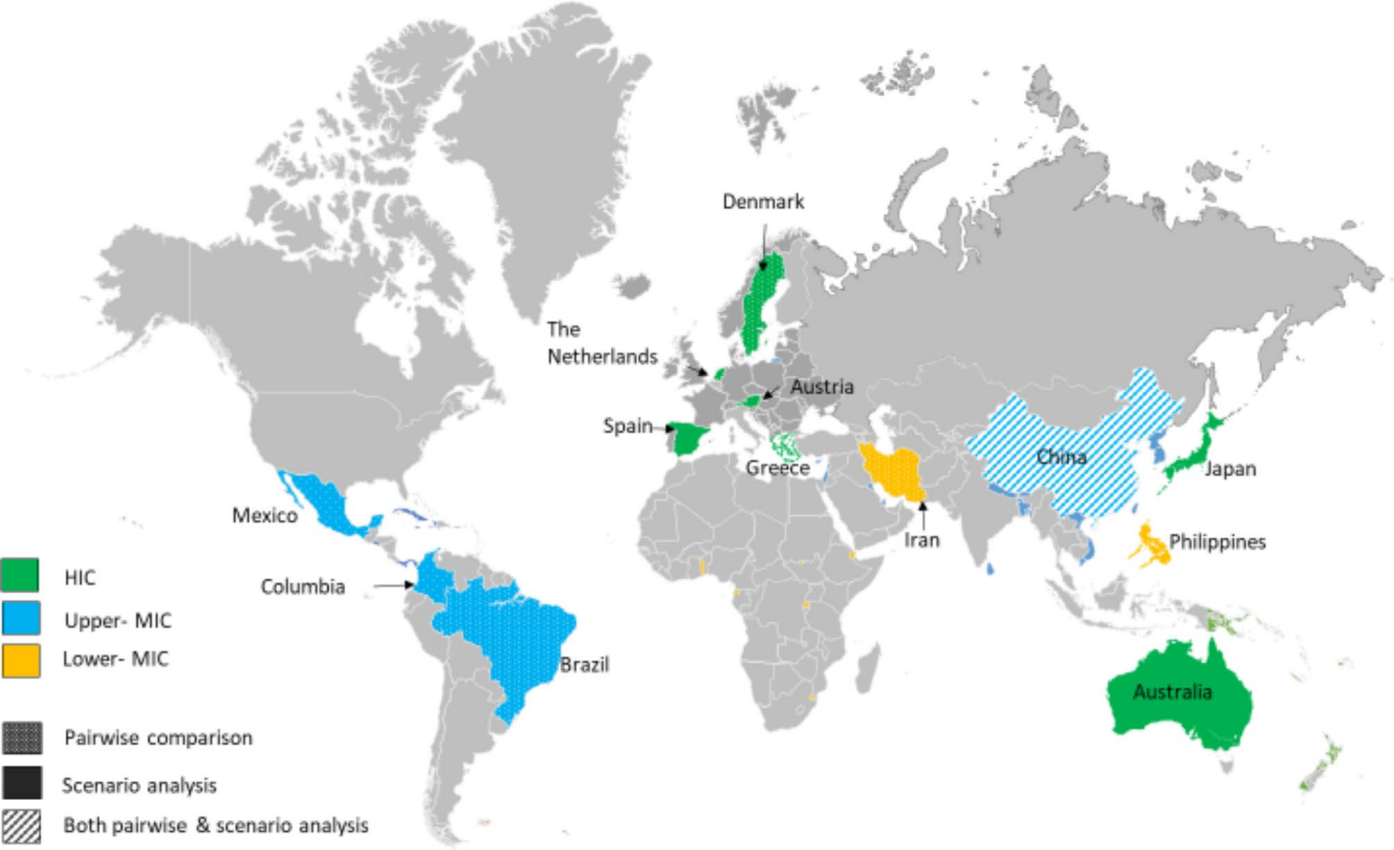
A map showing the different approaches for the 13 economic evaluation studies included in this review across regions. HIC, High Income Country; MIC, Middle Income Country. This figure was generated using the free editable map available at this link: https://www.pptmaps.com/Editable-map-of-the-world-with-country-borders.html^52^, and then used PowerPoint software Version 2019.

Table 1 presents the general characteristics of the 13 studies included. Of these, 7, 4 and 2 were conducted in HIC, upper-MICs, and lower-MICs, respectively as per the World Bank classification^42^, none were from low-income countries (LICs). Five studies were European^19,43–46^, three each from South-America^47–49^ and Western Pacific regions^10,20,50^, and a single study each from South-East Asia^18^ and the Eastern Mediterranean^11^, as presented in Fig. 2. Seven studies included CUAs^10,11,18,19,43,44,50^, five were both CUAs & CEAs^20,45–48^, and a single study was a CEA that reported outcomes in LYs gained^49^. All studies were in English except one reported in Spanish^48^.

**Table 1:** General characteristics of included studies.

	Study	Country	Region	Country income level	Economic evaluation type	Approach	Perspective	Time horizon (years)	Discount rate (costs and outcomes)
1.	Yang et al. [2021] ^10^	China	WPR	Upper-MIC	CUA	Pairwise & Scenario-based	Third-Party Payer	5, 10, 15	5%
2.	Bayani et al. [2021] ^18^	Philippines	SEAR	Lower-MIC	CUA	Scenario-based	Public Healthcare & Societal	Lifetime	3%
3.	Moradpour et al. [2020] ^11^	Iran	EMR	Lower-MIC	CUA	Pairwise	Societal	Lifetime	6%
4.	Rosselli et al. [2015] ^47^	Columbia	AMR	Upper-MIC	CUA&CEA	Pairwise	Third-Party Payer	1, 2, 3, 5	3%
5.	Jensen et al. [2014] ^43^	Denmark	EUR	HIC	CUA	Pairwise	Public Healthcare	6	3.5%
6.	Villa et al. [2012] ^19^	Spain	EUR	HIC	CUA	Scenario-based	Societal	5, 10, 15	3.5%
7.	Shimizu et al. [2012] ^50^	Japan	WPR	HIC	CUA	Scenario-based	Public Healthcare	15	3%
8.	Haller et al. [2011] ^46^	Austria	EUR	HIC	CUA&CEA	Scenario-based	Public Healthcare	10	3%
9.	Howard et al. [2009] ^20^	Australia	WPR	HIC	CUA&CEA	Scenario-based	Public Healthcare	5	5%
10.	Kontodimopoulos et al. [2008] ^44^	Greece	EUR	HIC	CUA	Pairwise	Public Healthcare	Lifetime	5%
11.	de Wit et al. [1998] ^45^	Netherlands	EUR	HIC	CUA&CEA	Scenario-based	Societal	5	5%
12.	Arredondo et al. [1998] ^48^	Mexico	AMR	Upper-MIC	CUA&CEA	Pairwise	Public Healthcare	5	5%
13.	Sesso et al. [1990] ^49^	Brazil	AMR	Upper-MIC	CEA	Pairwise	Third-Party Payer	2	No discounting

AMR, American Region; CEA, Cost-Effectiveness Analysis; CKD, Chronic Kidney Disease; CUA, Cost-Utility Analysis; EMR, Eastern Mediterranean Region; ESKD, End-stage kidney disease, EUR, European Region; HIC, High Income Country; MIC, Middle Income Country; SEAR, South-East Asia Region; UMIC, Upper-Middle Income Country; WPR, West Pacific Region.

Ten studies performed model-based analyses; two were cross-sectional, while one was a retrospective cohort that included an economic evaluation. Most studies reported a public-health provider perspective (*N* = 7); of the four studies that had a societal perspective, all included costs related to transportation while three reported indirect costs related to productivity loss. The time horizon in the studies ranged between 1 and lifetime, with most using a 5-year time horizon (*N* = 6). All but one study^49^ discounted for both health and outcomes, with the majority (*N* = 5) applying a 5% discount rate. Seven studies stated no conflict of interest^10,11,18,43,46,47,50^, and all but two^44,48^ declared their sources of funding. The information for country-specific ESKD prevalence was identified from independent reviews as it was not available for extraction from all of the thirteen studies included, which ranged from 0.03% in Columbia to 0.4% in Brazil.

### Model design

All 10 model-based designs were applied using Markov models, with one also incorporating decision tree analysis^43^. The states ranged from 4 to 36, with the majority having four states (*N* = 5). Four studies had the same four states i.e., HD, PD, KT and death^10,11,19,43^, whereas another study had a pre-emptive transplant state instead of PD^20^. Two studies had 8 and 12 states respectively with 1-month cycle lengths^46,47^. Only one^18^ study considered complications associated with HD and PD in its model states, after adoption from a previous study by Yot et al.^51^.

Individual studies generally included pairwise comparisons of KRTs, i.e., HD-PD, HD-KT and PD-KT^10,11,43,44,47–49^ to determine the most cost-effective modality or alternatively provided comparisons of various scenarios by varying proportions of patients receiving KRT^10,18–20,45,46,50^ that reflected the current KRT status in their respective countries. One study considered both approaches^10^. There were 24 comparison pairs in the pairwise approach and 36 in the scenario specific analysis resulting in 60 evaluations in total.

### Risk of bias assessment

Of the 13 studies, 7 addressed 60% of the ECOBIAS checklist items and were graded as low risk of bias. All studies measured resource use continuously, identified all costs relevant to ESKD and intervention, had an adequate comparator (i.e., usual care) and had transparent methods and process of data identification and data incorporation. Compliance with completeness of reporting according to the CHEERS checklist was 87% on average for all studies. No studies provided 100% reporting for all 28 items on the CHEERS checklist. The least reported items included characterizing distributional effects (23%), heterogeneity (31%) and conflict of interest (54%). These results are provided in Supplementary Tables S2 and S3 respectively.

### Cost-effectiveness results

#### Incremental cost effectiveness ratio

In the pairwise comparison approach, all studies reported KT as the most cost-effective option^10,11,15,43,44,47–49^. HD was considered to be more costly and less effective than PD in all studies except one^49^, where continuous ambulatory peritoneal dialysis (CAPD) was considered less cost-effective than HD.

In the scenario specific approach, Scenario 2, an increase in PD with a corresponding decrease in HD, was found to be most cost-effective in two studies^18,45^, while scenario 3, an increase in KT with a corresponding decrease in HD was found to be most cost-effective in two studies^20,50^. Scenario 4, an increase in both PD and KT with a decrease in HD, was found to be most cost-effective in two studies^10,46^ and lastly Scenario 5 was found to be most cost-effective in a single study^19^. These results are presented in Table 2.

**Table 2:** Cost-effectiveness findings of studies included.

	Author (Year of publication)	Comparator	Intervention	WTP-Threshold	Results	Estimated INB	Conclusion and recommendation
**Pairwise approach-based studies**							
1.	Yang et al. [2021] ^10^	HD	PD (5 years)	US$ 44,300	At a WTP-threshold of US$ 44,300, KT was most cost-effective compared to PD. At an ICER of US$ 35,518, PD was preferential to HD	14.68	The planning for KRT service delivery should incorporate efforts to increase the future utilization of KT and PD
		HD	PD (10 years)			24.05	
		HD	PD (15 years)			28.69	
		HD	KT (5 years)			75.04	
		HD	KT (10 years)			140.06	
		HD	KT (15 years)			188.40	
		PD	KT (5 years)			60.36	
		PD	KT (10 years)			116.01	
		PD	KT (15 years)			159.71	
2.	Moradpour et al. [2020] ^11^	HD	PD	US$ 12,400	KT is more cost-effective compared to PD (ICER US$ 1446 per QALY). PD was preferential to HD	−12.76	Efforts required to encourage living kidney donation and potential recruitment of brain-dead donors. Promote PD as a superior alternative to HD for eligible patients
		HD	KT			47.05	
		PD	KT			32.78	
3.	Rosselli et al. [2015] ^47^	HD	KT	US$ 20,000	KT was more cost-effective compared to dialysis from the second year (ICER US$ 11,788). It became the preferential alternative after the fourth year	27.33	Health systems should support programs that encourage KT over dialysis for patients with ESKD
4.	Jensen et al. [2014] ^43^	HD	KT	Not reported	KT was preferential as it yielded both lower costs (US$ 107,154 versus US$ 136,559) and better outcomes (4.4 QALY versus 1.7 QALY) compared to dialysis	446.06	KT should be prioritized over dialysis. Promotion to use kidneys from living donors
5.	Kontodimopoulos et al. [2008] ^44^	HD	PD	Not reported	KT was most cost-effective. The cost per QALY was higher in HD (US$ 87,342) compared to PD (US$ 78,877) and KT (US$ 65,880)	37.83	Initiation of a campaign to promote organ donation. After KT, promoting PD appears to be the next best option
		HD	KT			775.69	
		PD	KT			737.87	
6.	Arredondo et al. [1998] ^48^	HD	PD	Not reported	The most cost-effective intervention was KT (US$ 3088) followed by CAPD (US$ 6416) and HD (US$ 11,147)	26.40	Promotion of KT as the most cost-effective intervention for patients with ESKD
		HD	KT			50.50	
		PD	KT			24.10	
7.	Sesso et al. [1990] ^49^	HD	CAPD	Not reported	CAPD was less cost-effective than HD and both were less cost-effective than KT. The cost per year of survival was CAPD US$ 12,134, HD US$ 10,065, CD-KT US$ 6978 and LD-KT US$ 3022	−557.96	Although KT alternatives were reported to be more cost-effective, the dialysis alternatives had better survival rates
		CAPD	Cadaveric KT			−816.76	
		HD	Living Donor KT			−1701.61	
		HD	Cadaver KT			−1374.72	
**Scenario-based studies**							
1.	Yang et al. [2021] ^10^	Current Scenario 1: HD 73%, PD 14%, KT 13%	Scenario 2: Increase in incident patients on PD, i.e., HD 47%; PD 40%; KT 13%	US$ 44,300	Scenario 2 was preferential to scenario 1. Scenarios 3&4 were cost-effective (4 more than 3) compared with scenario 1. The results were consistent across three time-horizons; 5, 10 and 15 years	52,218.92	Increasing the proportion of incident patients on PD was preferential. Increasing the number of patients on both PD and KT resulted in an ICER below the threshold
			Scenario 3: Increase in incident patients on RT, i.e., HD 52%; PD 14%; KT 34%			213,211.30	
			Scenario 4: Increase in both PD and KT, i.e., HD 26%; PD 40%; KT 34%			265,431.67	
2.	Bayani et al. [2021] ^18^	Current scenario 1: 94% patients on HD—2 sessions/ week (90 sessions covered) 4% PD, 2% KT	Scenario 2: PD-first policy—11% HD, 87% of incident patients on PD, 2% KT.	US$ 7720.	All policy options were above the threshold, therefore not cost-effective. Scenario 2 (PD-first policy) had the least ICER (US$ 29,338), followed by Scenario 4 (US$ 29,747) then Scenario 5 (US$ 78,355)	−249.49	Shifting to a PD-first policy instead of expanding current HD coverage was the best strategy to make KRT affordable and sustainable for the health system
			Scenario 4: PD-first and pre-emptive transplant—No HD, PD 90% & the rest i.e., 10% are given KT upon diagnosis of ESKD.			−86.53	
			Scenario 5: Adequate HD—Expansion of HD coverage to 156 sessions/year to cover treatment thrice/ week, 4% PD, 2% KT.			−89.68	
3.	Villa et al. [2012] ^19^	Current situation of the Spanish KRT program: HD 46%, PD 5%, KT 49%	Scenario 2: increased proportion of scheduled patients on PD from 10–30%,	US$ 48,011	Scenario 1 was the least preferential. Scenarios 2 and 5b were the most cost-effective. The ICERs of scenarios 5a, 2, and 5b, compared with scenario 1, were US$ −114,060, US$ −486,936, and US$ −323,574 per QALY respectively	0.25	An increase in the overall scheduled incidence of KRT, and particularly that of PD, should be promoted
			Scenario 5a: increased proportion of overall scheduled incident patients from 57–75%			1.24	
			Scenario 5b: combined scenarios 2 and 3			1.27	
4.	Shimizu et al. [2012] ^50^	Current scenario 1 Base composition of KRT: 96.8% HD	Scenario 2: Likelihood of starting with PD increased by 2.3-times	US $50,000	Compared to the base scenario, the most cost-effective KRT was scenario 3b, followed by scenario 3a and 3c, (all three were preferential), then scenario 2	13.68	KT uptake should be promoted as more cost-effective
			Scenario 3a: Likelihood of a pre-emptive living donor transplant increased by 2.4-times			21.1	
			Scenario 3b: Likelihood of a living donor transplant increased by 2.4-times			36.03	
			Scenario 3c: Likelihood of a deceased donor transplant increased by 22-times			20.37	
5.	Haller et al. [2011] ^46^	Scenario 1: 90.6% of incident ESKD patients HD, PD 7.2%, LDKT 0.1%, DDKT 2.1%	Scenario 2: 20% of the incident ESKD patients were allocated to PD.	Not reported	Scenario 1 was less preferential to Scenario 2 & 4. Scenario 2 saved US$31 million and gained 839 QALYs; Scenario 4 saved US$46 million and gained 2242 QALYs	104,608.60	Live-donor KT is cost-effective and associated with increase in QALYs. Preemptive live donor KT should be promoted
			Scenario 4: 20% of incident ESKD patients were allocated to PD and additional 10% for preemptive KT from a living donor.			279,538.06	
6.	Howard et al. [2009] ^20^	Current scenario 1: Hospital HD 37.9%, Home HD 5.5%, PD 12.5%, KT 44.1%	Scenario 3a: annual incremental increase in KT to reach an extra 10% by 2010,	Not reported	Scenario 1 was less preferential to Scenario 3a & 3b. Scenario 2 was less costly and at least as effective. Increasing KT had a saving of US$4 million to US$20 million. Increasing PD had a net saving of US$94 million	19,999.87	KT increases survival and is most cost-effective. Moving people away from hospital-based to home-based dialysis is associated with lower costs
			Scenario 3b—annual incremental increase in KT to reach an extra 50% by 2010.			95,521.04	
7.	de Wit et al. [1998] ^45^	Scenario 1: base case—30 KT per million population + the other modalities	Scenario 2a: 10% of new CHD patients to CAPD	Not reported	When comparing dialysis modalities to each other, the ratio of cost/LY gained and cost/QALY was best for CAPD and worst for center-HD	46,627.09	KT and CAPD were the most cost-effective options, while center-HD was the least cost-effective option
			Scenario 2b: 20% of new CHD patients to CAPD			93,346.85	
			Scenario 3a: 38 KT per million population			87,503.85	
			Scenario 3b: 44 KT per million population			203,530.52	

Reclassification of scenarios to provide uniformity for cross study comparisons. Scenario 1—current status of KT modality coverage in each country was used as the comparator. Scenario 2—increase in PD coverage, decrease in HD, KT held constant. Scenario 3—increase in KT coverage, decrease in HD, PD held constant. Scenario 4—increase in both PD and KT, decrease in HD. Scenario 5—any other approach not captured under any of the aforementioned scenarios.

CAPD, Continuous ambulatory peritoneal dialysis; DDKT, Deceased donor kidney transplant; ESKD, End-stage kidney disease; HD, Haemodialysis; ICER, Incremental cost-effectiveness ratio; KT, Kidney Transplantation; LDKT, Living donor kidney transplantation; PD, Peritoneal dialysis; QALY, Quality-adjusted life-year; WTP, Willingness to pay.

Some studies also explored the various forms of KT (i.e., cadaver, and living donor transplant^46,49,50^, PD (i.e., CAPD, Automated PD; APD, or continuous cycling PD; CCPD)^45^; and HD (i.e., Center HD, Home HD, and limited care HD)^20,45^. In the comparison between the various forms of PD versus other HD modalities, CAPD was found to be the most cost-effective treatment modality, followed by home HD, then CCPD or APD; the least cost-effective was limited care HD i.e., in a non-hospital facility^45^.

Seven studies did not report WTP threshold; of those that did, three reported GDP-based thresholds^11,27,47^ and three country-specific thresholds^18,19,50^. Upon standardization to US$ 2022, the threshold value in the studies ranged from US$ 7720 to US$ 144,283.

### Cost-effectiveness (CE) plane

A CE plane was constructed for each comparison point with incremental QALYs on the X-axis and incremental costs on the Y-axis. This was presented separately for both approaches i.e., pairwise comparison of KRTs and scenario analysis with different population proportions under each modality.

**Fig. 3:**
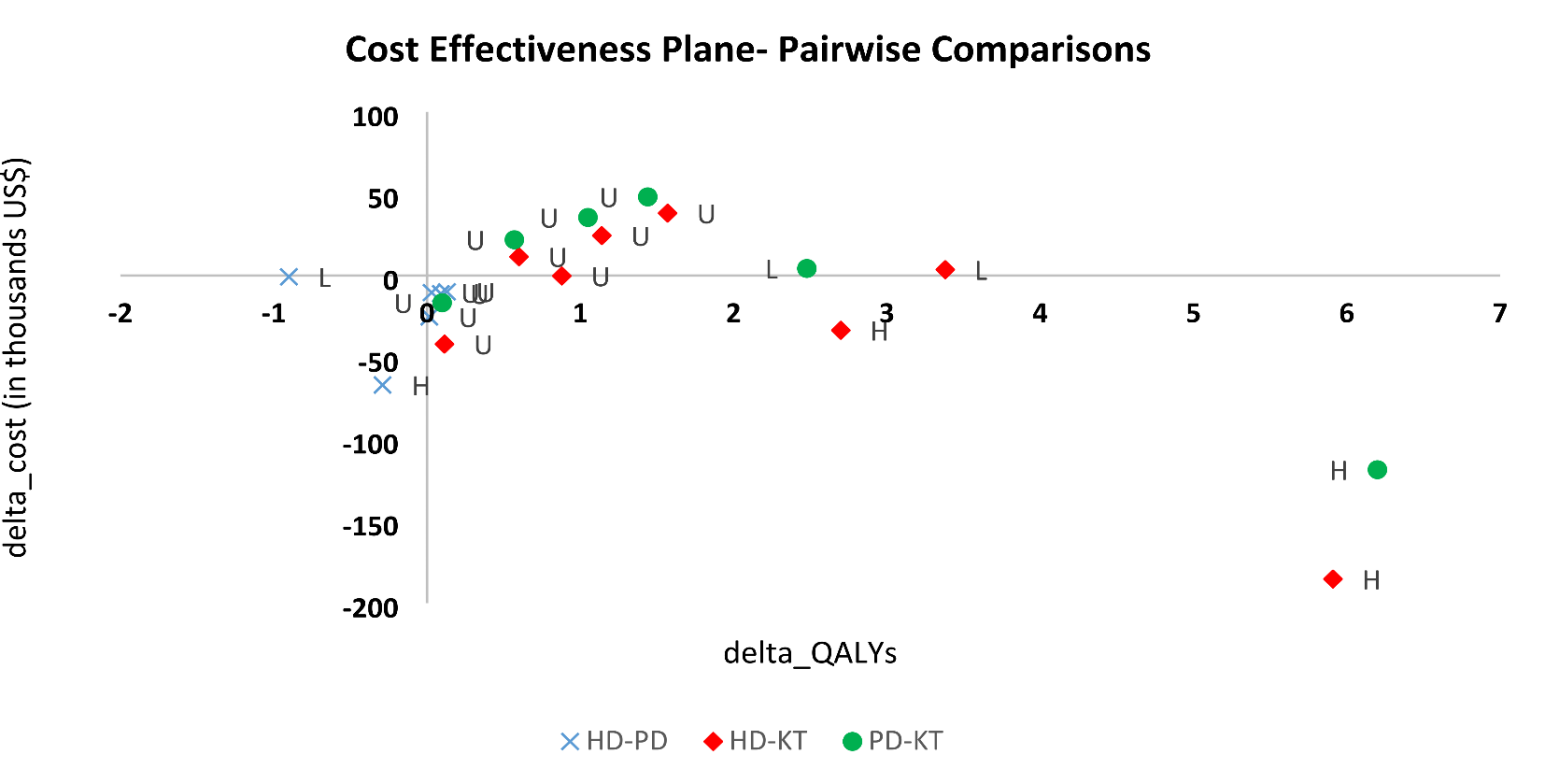
Cost-effectiveness plane for pairwise comparison approach. H, High income country; KT, Kidney Transplantation; L, Lower-middle income country; U, Upper-middle income country; HD, Haemodialysis; PD, Peritoneal Dialysis; QALY, Quality-Adjusted Life Year. The blue square-shape dots symbolize HD comparator & PD intervention; red diamond-shape dots, HD comparator & KT intervention and green round-shape dots, PD comparator & KT intervention.

The CE-plane of pairwise KRT comparisons demonstrated in Fig. 3 shows interventions were more effective than the comparators if they were presented in the right quadrant. Points located within the right-lower quadrant indicate that the intervention was dominant or preferential to the comparator, suggesting that most studies that had KT as the intervention were more likely to be cost-effective compared to either HD or PD, and these mostly originated from HICs and upper-MICs^43,44,48^. Those points within the right-upper quadrant (which means the intervention was more costly and also more effective than the comparator) all had KT as the intervention and either HD or PD as the comparator and originated from upper- and lower-MICs^10,11,47^. Two points located within the left-lower quadrant indicated that PD was less costly and less effective than HD representing two studies (one each from lower MIC^11^ and HIC^44^).

**Fig. 4:**
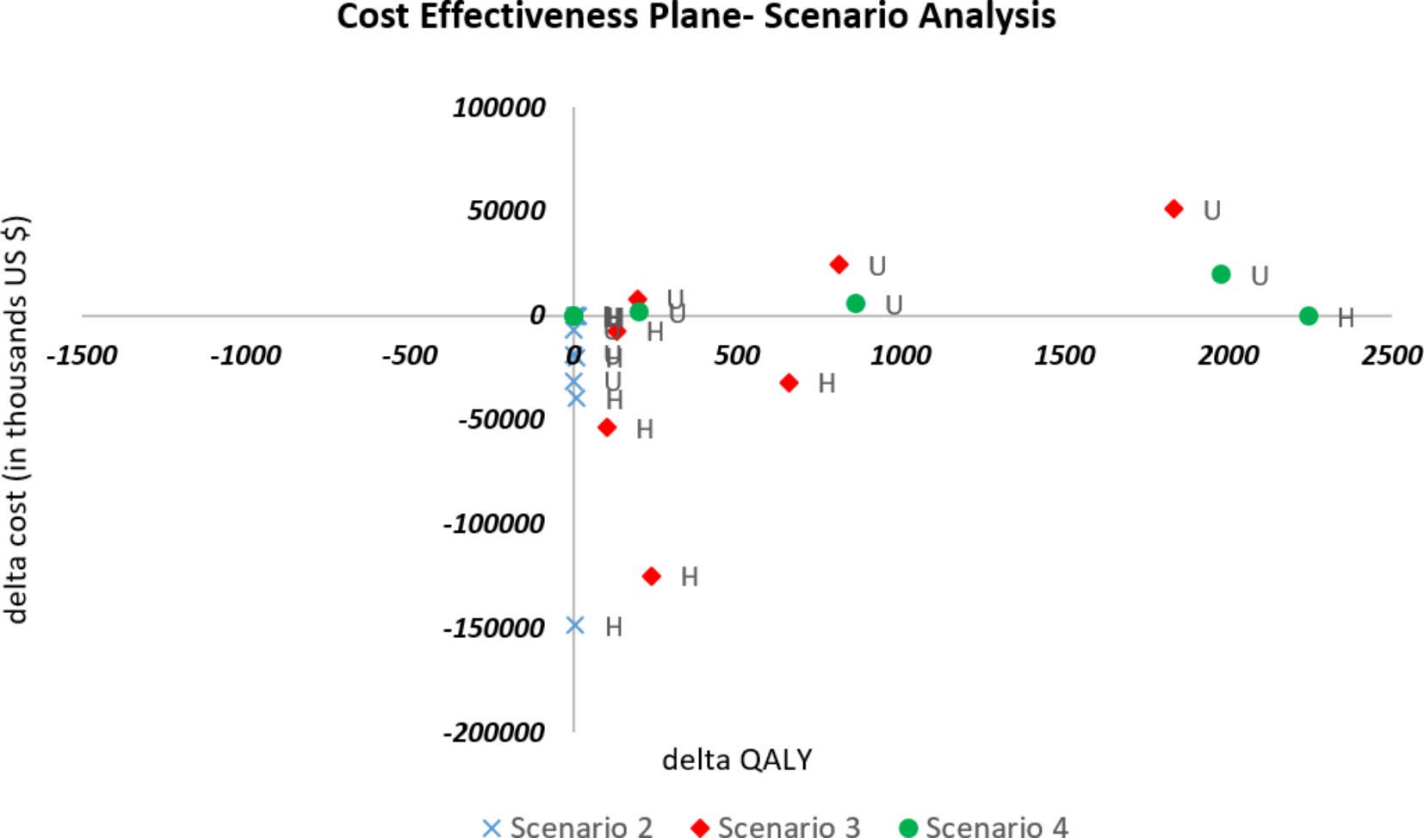
Cost-effectiveness plane for scenario analysis. H, High income country; L, Lower-middle income country; U, Upper-middle income country; QALY, Quality-Adjusted Life Year. The blue square-shape dots symbolize scenario 2; red diamond-shape dots scenario 3 and green round-shape dots scenario 4. All interventions compared against scenario 1.

In the CE-plane represented by the individual scenario analyses Fig. 4, all points were located in the right quadrant indicating that Scenarios 2–4 were more effective than Scenario 1 for each study. The figure also suggests that increased PD coverage and decreased HD coverage, with KT remaining constant (Scenario 2), was less costly and as effective or more effective than the current status^10,18–20,45,46,50^. Increased KT and decreased HD with PD remaining constant (Scenario 3), was more costly in upper-MICs^10^ in contrast to those from HICs^20,45,50^ which were less costly and cost-effective compared to the current status for that specific scenario. For Scenario 4, increased PD and KT/decreased HD, tended to be comparable or more costly than Scenario 1^10,18,46^; these would be cost-effective or not depending on country-specific WTP thresholds.

### Incremental net benefit (INB)

In the INB computation for the pairwise comparison of the KRTs, 5 of the 24 comparisons had negative INB, see Table 2. Thus, the intervention was not cost-effective relative to the comparator. It was also observed that the pair with HD as the comparator and KT as the intervention yielded the highest INB in each study. This was consistent despite differences in country income level, WTP-threshold, time horizon and perspective across all studies^10,11,27,44,47,48^.

For the scenario analysis approach, it was noted that one study^18^ yielded negative INB, indicating that all scenarios were not cost-effective compared to Scenario 1. This study was from a lower-MIC. All the other studies conducted in upper-MICs and HIC had positive INB. Scenario 4 had the highest INB overall, and included one study from upper-MIC and HIC^10,46^, signifying the greatest cost-effectiveness relative to threshold, compared to Scenario 1. This was followed by Scenario 3^20,45,50^.

## Discussion

This systematic review included thirteen studies to assess the cost-effectiveness of KRTs for ESKD by exploring two different approaches: pairwise comparison of KRT modalities and specific scenario analyses that reflected differential proportions for each KRT modality.

For the pairwise comparison approach, our findings suggested that KT tended to be the most cost-effective treatment option compared to HD and PD. PD was less costly than HD across all country income levels and offered comparable effectiveness. In the specific scenario approaches, increasing patient proportions for the provision of KT to between 12% and 34%, and PD to between 20% and 40%, while decreasing HD to between 26% and 68% provided the most cost-effective coverage for ESKD patients.

These findings prioritize KT and PD as the key strategies to enhance KRT sustainability in national health coverage systems, as is the current provision in many countries. Italy, the Netherlands and the United States of America (USA) have developed policies to increase kidney organ donation from both living and deceased donors^53,54^. Hong Kong and Thailand implement ‘PD-first’ policies for patients newly diagnosed with ESKD^55^. Canada, Spain, China, Taiwan and Mexico support PD-favored policies promoted through incentivizing PD uptake through increased reimbursement rates, training of personnel, and providing PD machines and supplies^56^. In Hong Kong, for example, the policy is to promote CAPD as the initial choice, while reserving APD for individuals with elevated membrane transfer levels or those with specific psychosocial requirements. APD machine subsidies are offered to promote this approach. During the COVID-19 pandemic, two health care reform initiatives were passed in the USA to increase support for home-based dialysis thereby reducing risk of exposure to the virus^57^. These policy-related changes have potential to tip the cost-effectiveness in favour of PD.

Although KT is considered the gold standard and most cost-effective modality, the promotion of KT remains the most challenging due to limited donor availability. Three studies explored incorporation of alternative forms of KT, i.e., cadaveric organ donation and pre-emptive KT. Though beneficial, these were less cost-effective compared to living-donor KT. Worldwide, the current KT requirement is 255 per million population. HICs especially from Eastern and Central Europe have greater coverage of KT at 363 pmp compared to upper-middle (80 pmp) and lower-middle (27 pmp) income countries from Africa and Latin America^9^.

As such, PD may represent a more feasible KRT for lower-MICs and LICs that face limitations due to infrastructural and professional capacity for performing KT. However, expanding PD, particularly in developing countries, may be limited due to the high cost of importing PD fluids and disposables^58^. Local manufacturing and volume-based negotiated pricing for these consumables are highly encouraged^56^. Additionally, there would be an institutional learning curve as the practice of PD in most of these countries is not widespread.

Nevertheless, despite the cost-effective benefits of KT and PD, the availability of HD within healthcare systems is especially important for patients with cardiovascular complications and those in need of intensive monitoring by healthcare professionals for better clinical outcomes^59^. Our findings suggest maintaining HD populations between 26 and 68% would be the most cost-effective option.

Quality assessment using the ECOBIAS checklist indicated that despite more than half of the studies included, having more than 60% of items graded as low risk of bias, the listing of studies in a trial register was not applicable to all studies and variable checking for double counting was unclear. Biases related to the model, information on internal consistency for mathematical logic was unclear for all studies, while consideration of all four principles of uncertainty, as well as synthesis of treatment effects using meta-analytic techniques, indicated that no studies were considered to have a low risk of bias.

Our study has several strengths. To the best of our knowledge, this is the first study to summarize cost-effectiveness of all strategies of KRT, and also consider specific scenarios for varying ESKD patient population proportions under each KRT modality. This was possible through the computation of INB instead of ICER as the economic effect measure, given the limitations associated with ICER calculation and interpretation. Despite the challenges of synthesizing economic evaluation studies, with varying levels of income, WTP thresholds, measurements of costs and utilities; stratification of these studies based on the approach used and converting monetary units to a common standard currency (US $2022), enabled indirect comparisons across both studies and countries. This study will be particularly important to LICs and lower-MICs, where cost is paramount to patients’ choice of dialysis modality.

Our study also had limitations. KRT cost-effectiveness findings may be less generalizable especially with lower-MICs and LICs, as the majority of studies included originated from HICs and upper-MICs. In addition, as data on measures of dispersion was not provided across all studies, pooling INB data by applying meta-analysis could not be undertaken, which represents a future opportunity. Our recommendations for economic evaluation studies would be to comprehensively report how costs were valued, the currency and year of conversion, as well as to report the mean values and the dispersions for the main categories of costs and outcomes of interest, to allow for comparability across studies.

## Conclusion

While KT was the most cost-effective KRT identified from the pairwise comparisons and an increase in the provision of both KT and PD coverage was supported from the specified scenario analyses, variability in cost-effectiveness of KRTs across country income levels was noted. These modalities were cost-effective in HIC and upper-MIC, but this was not applicable for lower-MICs and LICs. In addition, PD is a more cost-effective modality compared to HD, especially in regions where KT is not widely available. However, PD is often underutilized in lower-middle and low-income countries, which exacerbates the strain on healthcare budgets.

## Electronic supplementary material

Below is the link to the electronic supplementary material.

Supplementary Material 1

## Data Availability

The datasets used and/or analysed during the current study available upon request from the corresponding author.
