# Author Correction: A systematic review and quality assessment of economic evaluations of kidney replacement therapies in end-stage kidney disease

**DOI:** 10.1038/s41598-024-77061-x

**Published:** 2024-10-31

**Authors:** Patricia Nyokabi, Sitaporn Youngkong, Bhavani Shankara Bagepally, Tabitha Okech, Usa Chaikledkaew, Gareth J. McKay, John Attia, Ammarin Thakkinstian

**Affiliations:** 1 Mahidol University Health Technology Assessment Graduate Program, Bangkok, Thailand; 2 Ministry of Health, Nairobi, Kenya; 3 Social and Administrative Pharmacy Division, Department of Pharmacy, Faculty of Pharmacy, Mahidol University, Bangkok, Thailand; 4 Indian Council of Medical Research (ICMR), National Institute of Epidemiology, Chennai, India; 5 Centre for Public Health, Queen’s University Belfast, Belfast, UK; 6 Centre for Clinical Epidemiology and Biostatistics, School of Medicine and Public Health, University of Newcastle, Newcastle, NSW Australia; 7 Department of Clinical Epidemiology and Biostatistics, Faculty of Medicine, Ramathibodi Hospital, Mahidol University, Bangkok, Thailand

Correction to: *Scientific Reports *10.1038/s41598-024-73735-8, published online 03 October 2024

In the original version of this Article, Usa Chaikledkaew was omitted as a co-corresponding author. Correspondence and requests for materials should also be addressed to usa.chi@mahidol.ac.th.

The original Article has been corrected.

