# Supplementary material for: A systematic review and quality assessment of economic evaluations of kidney replacement therapies in end-stage kidney disease
Source: Sci Rep. 2024 Oct 3;14:23018. doi: 10.1038/s41598-024-73735-8 (PMC11450173; doi:10.1038/s41598-024-73735-8)
Supplement: Supplementary file 1 — Supplementary Material 1 [file 41598_2024_73735_MOESM1_ESM.docx]

**Supplementary**

**A systematic review and quality assessment of economic evaluations of kidney replacement therapies in end-stage kidney disease**

Patricia Nyokabi, Sitaporn Youngkong, Bhavani Shankara Bagepally, Tabitha Okech, Usa Chaikledkaew, Gareth J McKay, John Attia, Ammarin Thakkinstian

(All figures and tables in the supplementary appendices are created by the authors)

Supplementary Table S1: Electronic search strategies

- Overall Search Strategy

| **Domain** | **Search Terms** |
| --- | --- |
| Patient/Population (P) | “end stage renal disease” “end stage kidney disease” |
| Intervention (I) | “renal replacement therapy” “peritoneal dialysis” “hemodialysis” “haemodialysis” “kidney transplantation” “renal transplantation” |
| Outcome (O) | “quality adjusted life year” “disability-adjusted life year” “incremental cost effectiveness ratio” “incremental cost” “incremental effectiveness” |
| Study Design (S) | “economic evaluation,” “cost utility analysis,” “cost effectiveness analysis” “cost benefit analysis” |

- **Medline through PubMed (search date September 2022)**

| **DOMAIN** | **Number** | **Search Term** | **Search Results** |
| --- | --- | --- | --- |
| **P-I-S** | #33 | #4 AND #15 AND #30 | 2,408 |
| **P-I-O** | #32 | #4 AND #15 AND #23 | 665 |
| **P-I-O-S** | #31 | #4 AND #15 AND #23 AND #30 | 208 |
| **Overall S** | #30 | #24 OR #25 OR #26 OR #27 OR #28 OR #29 | 404,417 |
| **S** | #29 | 'economic evaluation' | 122,997 |
|  | #28 | 'cost effectiveness analysis' | 113,027 |
|  | #27 | 'cost effectiveness' | 146,792 |
|  | #26 | 'cost utility' | 284,023 |
|  | #25 | 'cost utility analysis' | 98,579 |
|  | #24 | cost benefit analysis[MeSH Terms] | 89,928 |
| **Overall O** | #23 | #16 OR #17 OR #18 OR #19 OR #20 OR #21 OR #22 | 56,205 |
| **O** | #22 | ICER | 5,539 |
|  | #21 | 'incremental cost effectiveness ratio' | 11,147 |
|  | #20 | 'incremental effectiveness' | 34,879 |
|  | #19 | 'incremental cost' | 19,242 |
|  | #18 | 'disability adjusted life year' | 5,327 |
|  | #17 | 'quality adjusted life year' | 24,750 |
|  | #16 | quality adjusted life year[MeSH Terms] | 14,857 |
| **Overall I** | #15 | #5 OR #6 OR #7 OR #8 OR #9 OR #10 OR #11 OR #12 OR #13 OR #14 | 357,945 |
| **I** | #14 | 'kidney transplantation' | 142,747 |
|  | #13 | kidney transplantation[MeSH Terms] | 102,673 |
|  | #12 | haemodialysis | 169,199 |
|  | #11 | renal dialysis[MeSH Terms] | 122,561 |
|  | #10 | dialysis | 208,349 |
|  | #9 | dialysis[MeSH Terms] | 145,403 |
|  | #8 | 'peritoneal dialysis' | 35,254 |
|  | #7 | peritoneal dialysis[MeSH Terms] | 27,648 |
|  | #6 | 'renal replacement therapy' | 237,622 |
|  | #5 | 'renal replacement therapy'[MeSH Terms] | 227,439 |
| **Overall P** | #4 | #1 OR #2 OR #3 | 140,292 |
| **P** | #3 | 'end stage renal disease' | 140,292 |
|  | #2 | end stage kidney disease[MeSH Terms] | 99,041 |
|  | #1 | end stage renal disease[MeSH Terms] | 99,041 |

- **Scopus database (search date September 2022)**

**Query string**

( ( TITLE-ABS-KEY ( 'end  AND  stage  AND  renal  AND  disease' ) )  OR  ( TITLE-ABS-KEY ( 'end  AND  stage  AND  kidney  AND  disease' ) ) )  AND  ( ( 'renal  AND  replacement  AND  therapy' )  OR  ( 'peritoneal  AND  dialysis' )  OR  ( 'hemodialysis' )  OR  ( ( 'kidney  AND  transplantation' )  OR  ( 'renal  AND  transplantation' ) ) ) AND  ( ( 'quality  AND  adjusted  AND  life  AND  year' )  OR  ( 'disability  AND  adjusted  AND  life  AND  year' )  OR  ( 'incremental  AND  cost  AND  effectiveness  AND  ratio' )  OR  ( 'incremental  AND  cost' )  OR  ( 'incremental  AND  effectiveness' ) )  AND  ( ( 'economic  AND  evaluation' )  OR ( 'cost  AND  utility  AND  analysis' )  OR  ( 'cost  AND benefit  AND  analysis' ) OR  ( 'cost  AND  effectiveness  AND  analysis' ) )

| DOMAIN | SEARCH TERM | RESULTS |
| --- | --- | --- |
| Overall | P-I-S | 5105 |
| Overall | P-I-O | 1254 |
| Overall | P-I-O-S | 597 |

- **Embase database (search date September 2022)**

| **DOMAIN** | **SEARCH TERM** | **RESULTS** |
| --- | --- | --- |
| Overall | P-I-S | 178 |
| Overall | P-I-O | 98 |
| Overall | P-I-O-S | 79 |
| S | 'cost effectiveness analysis‘/exp |  |
|  | 'cost utility analysis‘/exp |  |
|  | 'cost benefit analysis‘/exp |  |
|  | 'economic evaluation‘/exp |  |
| O | 'incremental cost effectiveness ratio‘/exp |  |
|  | 'disability-adjusted life year‘/exp |  |
|  | 'quality adjusted life year‘/exp |  |
| I | 'kidney transplantation‘/exp |  |
|  | 'peritoneal dialysis‘/exp |  |
|  | 'hemodialysis‘/exp |  |
|  | 'renal replacement therapy‘/exp |  |
| P | 'end stage renal disease'/exp |  |

- **CEA Tufts Registry (search date September 2022)**

Keywords ‘end-stage renal disease’ and ‘renal replacement therapy’ were combined.

Result- 14 studies

- **NHS-EED, DARE, HTA Database**

Keywords ‘end-stage renal disease’ and ‘economic evaluation’ and ‘renal replacement therapy” were used.

Result- 10 studies

Supplementary Table S2: Risk of bias assessment by ECOBIAS

|  |  | No | Yang et al., 2021 | Moradpour et al., 2020 | Bayani et al., 2021 | Diego Rosselli | Haller et al., 2011 | Howard et al., 2009 | Shimizu et al., 2012 | de Wit et al., 1998 | Arredondo et al. 1998 | Kontodimopoulos et al., 2008 | Jensen et al., 2014 | Villa et al., 2012 | Sesso et al., 1990 |
| --- | --- | --- | --- | --- | --- | --- | --- | --- | --- | --- | --- | --- | --- | --- | --- |
| **Part A Overall checklist for bias in economic evaluation** | Was a societal perspective adopted? If not, has a different perspective been justified? | 1 | P | Y | Y | P | Y | Y | P | Y | P | Y | P | Y | Y |
|  | Was the best alternative chosen as comparator? Was current practice chosen as a comparator? Have all comparators been described in sufficient detail? | 2 | Y | P | Y | Y | Y | Y | Y | Y | Y | Y | Y | Y | Y |
|  | Were all costs relevant to the disease and intervention identified and considered? | 3 | Y | Y | Y | Y | Y | Y | Y | Y | Y | Y | Y | Y | Y |
|  | Was the resource use measured continuously? | 4 | Y | Y | Y | Y | Y | Y | Y | Y | Y | Y | Y | Y | Y |
|  | Is the price calculation presented in a detailed manner? Have reference prices been used? | 5 | Y | N | Y | Y | Y | Y | Y | Y | Y | Y | Y | Y | Y |
|  | Have ordinal scales for the outcomes measure in a CEA been used? | 6 | Y | Y | Y | P | Y | Y | Y | Y | Y | Y | N | Y | Y |
|  | Are variables adequately checked for double counting? | 7 | U | U | U | U | U | U | U | U | U | U | U | U | U |
|  | Have discounting rates from guidelines been applied? | 8 | Y | Y | Y | Y | Y | Y | Y | Y | Y | Y | Y | Y | N |
|  | Have the four principles of uncertainty (methodological, structural, heterogeneity, parameter) been considered in sufficient detail? | 9 | P | P | P | P | P | P | P | P | N | P | P | P | P |
|  | Have sponsorships been disclosed? Is the study protocol freely accessible? | 10 | Y | Y | Y | Y | Y | Y | Y | Y | N | N | P | Y | N |
|  | Has the study/trial been listed in a trial register? Have all results been reported according to the study protocol? | 11 | NA | NA | NA | NA | NA | NA | NA | NA | NA | NA | NA | NA | NA |
| **Part B Model-specific aspects of bias in economic evaluation** | Is the model structure in line with coherent theory? Do treatment pathways reflect the nature of disease? | 12 | Y | Y | Y | Y | Y | Y | Y | Y | NA | NA | P | Y | NA |
|  | Is there an adequate comparator, i.e., care as usual? | 13 | Y | Y | Y | Y | Y | Y | Y | Y | Y | Y | Y | Y | Y |
|  | Is the model chosen adequate regarding the decision problem? | 14 | Y | Y | Y | Y | Y | Y | Y | Y | NA | NA | P | Y | NA |
|  | Was a lifetime horizon chosen? Were shorter time horizons adequately justified? | 15 | N | Y | Y | P | Y | Y | Y | Y | N | Y | N | P | P |
|  | Are the methods of data identification transparent? Are all choices justified adequately? Do the input parameters come from high- quality and well-designed studies? | 16 | Y | Y | Y | Y | Y | Y | Y | Y | Y | Y | Y | Y | Y |
|  | Are probabilities, for example, based on natural history data? Is transformation of rates into transition probabilities done accurately? | 17 | Y | U | Y | Y | Y | Y | Y | Y | NA | NA | U | U | NA |
|  | Are relative treatment effects synthesized using appropriate meta- analytic techniques? Are extrapolations documented and well justified? Are alternative assumptions explored regarding extrapolation? | 18 | P | U | P | P | P | P | P | P | U | P | P | P | U |
|  | Are the utilities incorporated appropriate for the specific decision problem? | 19 | Y | Y | Y | Y | Y | Y | Y | Y | Y | Y | Y | Y | NA |
|  | Is the process of data incorporation transparent? Are all data and their sources described in detail? | 20 | Y | Y | Y | Y | Y | Y | Y | Y | Y | Y | Y | Y | Y |
|  | Have the four principles of uncertainty (methodological, structural, heterogeneity, parameter) been considered? | 21 | P | P | P | P | P | P | P | P | N | P | P | P | P |
|  | Has internal consistency in terms of mathematical logic been evaluated? | 22 | U | U | U | U | U | U | U | U | U | U | U | U | U |

N, No – it is high risk of bias; NA, Not Applicable; P, Partial; U, Unclear; Y, Yes – It is low risk of bias

**Supplementary Table S3: Risk of bias assessment by CHEERS Checklist**

Graph showing proportion of studies complying with CHEERS Checklist

**Supplementary Table S4: Currency Conversion**

|  | **currency year** | **cpi (conversion to 2022)** | **ppp (conversion to US $)** |
| --- | --- | --- | --- |
| Yang et al. | 2021 | 1.085655939 | 1 |
| Moradpour et al. | 2020 | 1.21 | 1 |
| Bayani et al. | 2021 | 1.120703297 | 0.0515 |
| Rosselli et al. | 2015 | 1.538424876 | 1 |
| Haller et al. | 2011 | 1.332082637 | 1.200480192 |
| Howard et al. | 2009 | 1.573123067 | 0.773395205 |
| Shimizu et al. | 2012 | 1.07296599 | 1 |
| de Wit et al. | 1998 | 1.756589833 | 1.199040767 |
| Arredondo et al. | 1998 | 6.326765358 | 1 |
| Kontodimopoulos et al. | 2008 | 1.403991514 | 1.447178003 |
| Jensen et al. | 2014 | 1.136672463 | 0.132205182 |
| Villa et al. | 2012 | 1.240955063 | 1.371742112 |
| Sesso et al. | 1990 | 2.76 | 1 |

Table showing the currency conversions
